# Assessment of Metabolic Syndrome in Patients with Chronic Obstructive Pulmonary Disease: A 6-Month Follow-Up Study

**DOI:** 10.3390/diagnostics14212437

**Published:** 2024-10-31

**Authors:** Elena-Andreea Moaleș, Lucia Corina Dima-Cozma, Doina-Clementina Cojocaru, Ioana Mădălina Zota, Cristina Mihaela Ghiciuc, Cristina Andreea Adam, Mitică Ciorpac, Ivona Maria Tudorancea, Florin Dumitru Petrariu, Maria-Magdalena Leon, Romică Sebastian Cozma, Florin Mitu

**Affiliations:** 1 Department of Medical Specialities I, Faculty of Medicine, “Grigore T. Popa” University of Medicine and Pharmacy, University Street No. 16, 700115 Iași, Romaniacdimacozma@yahoo.com (L.C.D.-C.); ioana-madalina.chiorescu@umfiasi.ro (I.M.Z.); mitu.florin@yahoo.com (F.M.); 2 Clinical Rehabilitation Hospital, Pantelimon Halipa Street No. 14, 700661 Iași, Romania; 3 Pharmacology, Clinical Pharmacology and Algeziology, Department of Morpho-Functional Sciences II, Faculty of Medicine, “Grigore T. Popa” University of Medicine and Pharmacy, University Street No. 16, 700115 Iași, Romania; 4 Saint Mary Emergency Children Hospital, 700887 Iași, Romania; 5 Advanced Research and Development Center for Experimental Medicine “Prof. Ostin C. Mungiu”—CEMEX, “Grigore T. Popa” University of Medicine and Pharmacy, 700115 Iași, Romaniatudorancea.ivona.maria@email.umfiasi.ro (I.M.T.); 6 Department of Preventive Medicine and Interdisciplinarity, Faculty of Medicine, “Grigore T. Popa” University of Medicine and Pharmacy, University Street No. 16, 700115 Iași, Romania; 7 Department of Otorhinolaryngology, Faculty of Medicine, “Grigore T. Popa” University of Medicine and Pharmacy, University Street No 16, 700115 Iași, Romania; 8 Romanian Academy of Medical Sciences, 030167 Bucharest, Romania; 9 Romanian Academy of Scientists, 050045 Bucharest, Romania

**Keywords:** chronic obstructive pulmonary disease, metabolic syndrome, self-management, quality of life

## Abstract

Background/Objectives: The association between chronic obstructive pulmonary disease (COPD) and metabolic syndrome (MetS) is a common one, with long-term therapeutic and prognostic impact. In view of the high pulmonary and cardiovascular morbidity and mortality, self-management contributes to decreasing the risk of an acute cardiac event or pulmonary decompensation. Methods: We conducted a prospective cohort study on 100 patients admitted to Iasi Clinical Rehabilitation Hospital who were divided into two groups according to the presence (67 patients) or absence (33 patients) of MetS. All patients benefited from multidisciplinary counseling sessions on their active role in improving modifiable cardiovascular risk factors and thus increasing quality of life. The aim of this study was to examine the impact of metabolic syndrome on lung function and the role of self-management in a 6-month follow-up period. The demographic, anthropometric, cardiovascular risk factors, and respiratory function were analyzed at baseline and at 6 months. Results: The presence of MetS was associated with higher fasting blood glucose (*p* = 0.004) and triglycerides (*p* = 0.003) but not with higher levels of interleukins or TNF-alpha. At the 6-month follow-up, abdominal circumference, forced expiratory volume in one second (FEV1), dyspnea severity, and blood pressure values improved in male patients with COPD. Systolic and diastolic blood pressure decreased in the COPD group as a whole, but especially in male patients with and without associated MetS. BMI was positively correlated with FEV1 (r = 0.389, *p* = 0.001) and the FEV1/forced vital capacity (FVC) ratio (r = 0.508, *p* < 0.001) in all COPD patients and in the MetS subgroup. In the COPD group as a whole. the six-minute walk test (6MWT) results (m) were positively correlated with FEV1 and FVC. The correlation remained significant for FVC in COPD patients with and without MetS. An increase in BMI by one unit led to an increase in TG values by 3.358 mg/dL, and the presence of metabolic syndrome led to an increase in TG values by 17.433 mg/dL. Conclusions: In our study, MetS is a common comorbidity in patients with COPD and is associated with higher BMI, fasting glucose, and triglycerides but not with the inflammatory parameters. A mixed pulmonary–cardiovascular rehabilitation intervention leads to improvement in various parameters in both female and male COPD patients.

## 1. Introduction

Chronic obstructive pulmonary disease (COPD) is a heterogeneous condition, with a major impact on the population in terms of the association between pulmonary and cardiovascular complications [1]. The symptoms of patients with COPD are non-specific, represented by dyspnea, chronic cough, or sputum production [2,3]. Spirometry confirms the diagnosis of COPD by recording a post-bronchodilator ratio of forced expiratory volume in one second (FEV1)/forced vital capacity (FVC) less than 70% [4]. However, there are subjects with a FEV1/FVC ≥ 0.7 ratio after bronchodilator, but with physiological alterations such as altered FEV1 (low or rapidly declining FEV1), lung hyperinflation, or gas trapping [5,6]. All these changes place patients in the “Pre-COPD” stage and require changing modifiable factors such as smoking cessation and cessation of exposure to respiratory emissions [7,8,9]. Thus, COPD can be prevented and treated, but most of the time, the disease is underdiagnosed, and patients receive inadequate treatment [10,11]. COPD does not only affect the lungs; it is a systemic disease [12,13,14], with the associated comorbidities contributing to the increase in mortality regardless of the COPD stage [15].

According to the recent criteria proposed by the International Diabetes Federation for the diagnosis of Metabolic syndrome (MetS) [16], MetS is a cluster of at least three clinical and/or biological markers (dysglycemia, elevated blood pressure, dyslipidemia, and central adiposity) associated with a pro-inflammatory phenotype, increased cardiovascular risk, and all-cause mortality. The prevalence of MetS depends on population aging, race, gender, presence of obesity (particularly central obesity), and history of diabetes or other comorbidities [17]. Although the pathophysiology of MetS is complex, the following mechanisms increase the risk of developing cardiovascular complications: visceral adiposity and insulin resistance [18]. Previous research has shown that patients with metabolic syndrome (MetS) are three times more likely to experience a stroke or heart attack and twice as likely to die from these conditions compared with those without MetS [19]. MetS is considered a persistent condition resulting from the intricate interplay between environmental and genetic factors [20]. Heritability estimates for MetS range from about 10% to 30% [19]. Additionally, environmental influences like lack of physical activity, poor diet, stress, and tobacco use are strongly linked to the development of MetS [21]. Because of the ongoing obesity pandemic, the incidence of MetS and COPD has been on the rise. In fact, patients with COPD often associate metabolic syndrome (MetS), but the mechanism is not well known [1,22,23]. Although smoking is a common risk factor, multiple studies do not mention a causal link between COPD and the occurrence of MetS [24]. Concomitant COPD and MetS lead to cardiovascular complications through increased atherogenic risk [25]. Some studies have shown that the presence of MetS in COPD patients worsens respiratory symptoms and lung function because of the presence of chronic systemic inflammation [26,27,28]. Declining lung function is indicated by a reduction in FEV1, a high degree of dyspnea, a decreased distance on the 6-minute walk test, and, implicitly, an increase in the dose of inhaled therapy [29,30].

**Figure 1:**
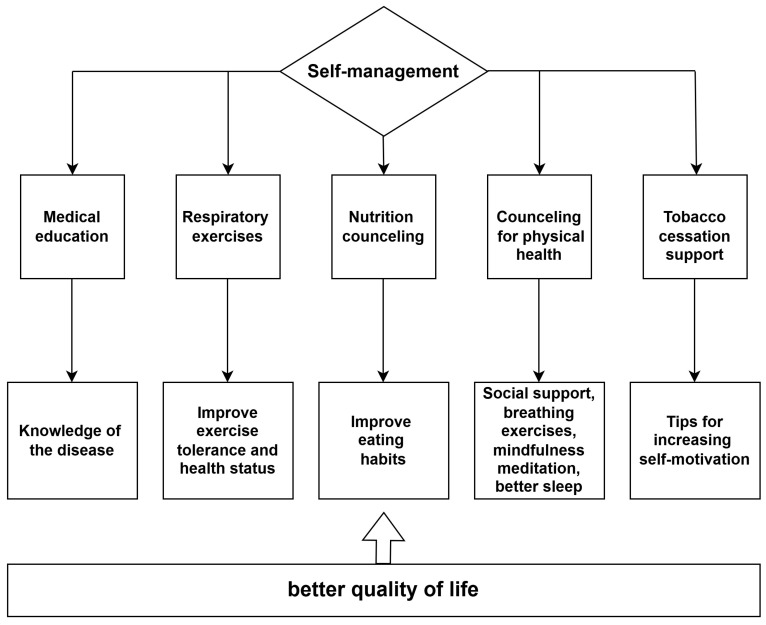
The importance of self-management in relation to the quality of life in patients with COPD.

Recent studies report a high frequency of the presence of MetS in young patients with COPD and less severe forms of the disease [31,32,33]. Considering this aspect, special attention is required in the evaluation of these patients. Moreover, good self-management of the disease can improve the prognosis and decrease the risk of cardiovascular morbi-mortality [34,35,36] (Figure 1).

However, the body mass index (BMI) is an inaccurate indicator of disease prognosis, as data from the literature show an increase in mortality among COPD subjects with low BMI and low muscle mass [37,38]. Abdominal obesity, a criterion for diagnosis of metabolic syndrome, negatively influences lung function [39]. Smokers have an increased risk of abdominal obesity and, implicitly, a high degree of systemic inflammation [40,41].

The aim of this study was to examine the impact of metabolic syndrome on lung function and the role of self-management in a 6-month follow-up period. The demographic, anthropometric, cardiovascular risk factors, and respiratory function were analyzed at the baseline and at 6 months.

## 2. Materials and Methods

### 2.1. Study Design

We conducted a prospective study of 127 consecutive patients with stable COPD admitted to the Pulmonary Rehabilitation Clinic and Cardiovascular Rehabilitation Clinic, Iasi Clinical Rehabilitation Hospital, over a period of 18 months (January 2022 and June 2023). Twenty-seven patients did not present for follow-up evaluation (after 6 months) and, in the absence of medical data, were excluded from the study group, so the final group of patients included a total of 100 subjects.

The inclusion criteria were as follows: a defined diagnosis of COPD (according to the GOLD guidelines) [42], the age of patients over 35 years old, and those who consented to participate in this study. Patients with malignancies, major neurologic and neuropsychiatric disorders, patients with kidney and liver failure (in the context of the interaction with interleukin values and the potential increasing effect on serum interleukin levels), or patients who refused to sign the informed consent were excluded from this study. All patients had a negative COVID-19 real-time reverse transcription PCR (RT-PCR) upon admission.

### 2.2. Measurements

In our study, we included demographic, anthropometric, biological, and functional parameters. The clinical and paraclinical parameters were evaluated both at enrollment and 6 months after the start of integrative, multidisciplinary management.

#### 2.2.1. Comorbidities

The initial evaluation was focused on the identification of comorbidities and cardiovascular risk factors with a role in the diagnostic and therapeutic strategy and implicitly with long-term prognostic value. Diabetes mellitus [43], dyslipidemia [44], hypertension [45], atrial fibrillation [46], and cardiovascular pathologies (chronic coronary syndrome [47], heart failure [48], peripheral vascular disease [49], or pulmonary hypertension [50]) were diagnosed based on the definitions of clinical guidelines or via previous medical records.

Among the associated lung diseases, obstructive sleep apnea syndrome with chronic oxygen demand was identified. Depression and anxiety completed the list of psychiatric comorbidities in the study group.

#### 2.2.2. Anthropometric Data, Symptoms and Medication

The body mass index (BMI) was calculated as the ratio of weight (kg) and height (m^2^). Dyspnea was assessed through the modified Medical Research Council scale (mMRC) and the Borg scale [51,52].

The COPD Assessment Test (CAT) score [53,54] was obtained using an eight-item response theory as follows: the first two questions were related to symptoms like cough and sputum, and the other six questions were about dyspnea, limitations of activity, and self-confidence. Each item had maximum of 5 points (0–5 points), and the maximum total score was 40 points (0–40 points).

As part of the multidisciplinary management to improve cardiovascular risk factors, patients enrolled in this study were treated with hypolipidemic (statins and fenofibrate), hypoglycemic (oral antidiabetics), insulin or antihypertensive (angiotensin-converting enzyme inhibitors [ACEIs], sartans, or diuretics) agents. A significant percentage of patients also received treatment with beta-blockers or drugs for psychiatric disorders.

#### 2.2.3. Metabolic Syndrome

The diagnosis of MetS was based on the same individual meeting at least 3 of the following criteria specified by the International Diabetes Federation [16]:

- Abdominal circumference ≥ 94 cm in men and ≥80 cm in women;
- Serum triglyceride value ≥ 1.7 mmol/L (150 mg/dL) or specific treatment for hypertriglyceridemia according to the clinical guidelines;
- High-density lipoprotein cholesterol (HDL) cholesterol: males < 1.03 mmol/L (40 mg/dL), females < 1.3 mmol/L (50 mg/dL) (or specific treatment for hyperlipidemia according to the clinical guidelines);
- Systolic blood pressure (BP) ≥ 130 mmHg and/or diastolic BP ≥ 85 mmHg or antihypertensive treatment according to the clinical guidelines;
- Fasting plasma glucose ≥ 5.6 mmol/L (100 mg/dL) or diagnosis of type 2 diabetes mellitus according to the clinical guidelines.

#### 2.2.4. Laboratory Data

Biological samples were collected both at enrollment and 6 months following the first evaluation. Blood count, lipid profile (low-density lipoprotein cholesterol (LDL), HDL, triglycerides), serum fasting glucose, and special markers of systemic inflammation (interleukin (IL)-8, IL-6, IL-1 beta (IL-1β), tumor necrosis factor (TNF)-alpha) completed the investigation of the proinflammatory status. Special biomarker (IL-8, IL-6, IL-1β, TNF-alpha) levels were measured in human serum, using commercial ELISA kits according to the manufacturer’s protocols, at the Advanced Research and Development Center for Experimental Medicine ”Prof. Ostin C. Mungiu”—CEMEX, Iasi, Romania.

The range of values considered to be within normal limits was less than 7 pg/mL for IL-6, less than 15 pg/mL for IL-8, less than 5 pg/mL for IL-1β and less than 8.1 pg/mL for TNF-alpha. The results were presented according to the International System of Units. 

#### 2.2.5. Pulmonary Function

Spirometry was performed in all patients included in this study to assess lung function. Per current professional society criteria, the measurements were taken by an experienced examiner, with a minimum of three valid measurements per subject. The forced expiratory volume in the first second [FEV1], FVC, FEV1/FVC ratio, and the maximal expiratory flow at 50% of the FVC [MEF50] were significant spirometry metrics that were taken into consideration. Body temperature, ambient barometric pressure, saturated with water vapor [BTPS], and the patients’ birth sex, ethnicity, age, height, and weight were recorded in order to calculate the predicted values for all spirometry outcomes. According to current guidelines [55,56], normal values were defined as FEV1/FVC ratios greater than 70% and FEV1, FVC, and MEF50 greater than 80% of their predicted values.

According to the Global Initiative for Chronic Obstructive Lung Disease (GOLD), airflow limitation was defined as an FEV1/FVC ratio less than 70%. COPD severity was defined into 4 grades as follows: stage I: FEV1 ≥ 80%, stage II: 80% > FEV1 ≥ 50%, stage III: 50% > FEV1 ≥ 30%, and stage IV: FEV1 < 30%.

#### 2.2.6. The 6 Min Walk Test (6MWT)

According to ATS/ERS [57,58], the 6-minute walk test (6MWT) was performed on all patients who were enrolled in the trial in order to evaluate their ability to exercise. Waist, weight, and previously administered medicine (type, dose) were measured before the test. The number of laps (full and half final laps) was counted in a 30 m level corridor. Before and after the test, the following parameters were measured: test duration, heart rate, blood pressure, dyspnea (measured using the Borg scale), and SpO_2_. During the examination, angina pectoris, vertigo, and varying degrees of lower limb discomfort (hip, calf, leg) were the primary complaints observed.

#### 2.2.7. Multidisciplinary Management

The patients included in this study benefited from integrative, multidisciplinary management that focused on the correction of modifiable cardiovascular risk factors (correction of dyslipidemia and diabetes according to ESC guidelines [43], normalization of BP profile, weight management), dietary counseling, psychotherapy, smoking cessation, and physical exercise.

### 2.3. Statistical Analysis

The descriptive statistics were calculated using the Statistical Package for the Social Science (SPSS) statistics software (version 27.0 for Windows, SPSS Inc., Chicago, IL, USA) for statistical analysis. The results were presented as mean ± standard deviation (SD) or as percentages (%) for categorical variables. The Kolmogorov–Smirnov test was used to assess the normal distribution of the data. Continuous variables were compared using the *t*-test (parametric analysis). Categorical variables were compared using the Fisher exact test. Pearson’s and Spearman’s (r) correlation coefficients were used to test the reliability of statistically significant correlations identified in our study. To assess the role of reducing serum triglycerides on the cardiovascular profile at a 6-month follow-up, a regression analysis was performed. A *p*-value of ≤0.05 was considered to be statistically significant.

### 2.4. Ethics

The protocol of this study was approved by both the Ethics Research Committee of the “Grigore T. Popa” University of Medicine and Pharmacy, Iasi, Romania, nr. 122, on 11 November 2021, and the Ethics Committee of the Iasi Clinical Rehabilitation Hospital, Romania, on 10 December 2021.

All patients signed an informed consent form, which stated that their medical records would be used for research purposes only.

## 3. Results

We analyzed 100 COPD patients who were divided into two groups according to the presence of MetS as follows: a group of 67 patients with MetS and another with 33 patients without MetS. The main demographic, anthropometric characteristics, comorbidities, biological data, and medications administered are presented in Table 1. The mean age of the patients was slightly higher in the second group of patients, without statistical significance (*p* = 0.155). In both groups, male patients were predominantly included (62.7% vs. 78.8%, *p* = 0.105).

Regarding anthropometric data, the mean BMI was significantly higher in the group of patients with COPD and MetS (32.57 ± 6.635 vs. 26.68 ± 5.488, *p* < 0.001). Also, the percentage of patients with BMI above the upper limit of normal was higher in the first group, being statistically significant in our study group.

Both groups included predominantly patients with COPD of medium severity (52.2% vs. 57.6%).

Regarding biological markers, serum blood glucose (*p* = 0.004) and serum triglycerides (*p* = 0.003) were higher in patients with COPD and MetS. No statistically significant differences were reported regarding LDL, HDL, serum levels of interleukins, or TNF-alpha.

Current treatment with lipid-lowering medication (especially statins—*p* = 0.013), as well as oral antidiabetic drugs (*p* = 0.002, except insulin), was more prevalent in the MetS subgroup.

Table 2 illustrates gender differences regarding associated comorbidities in the two subgroups. Diabetes mellitus and dyslipidemia were more prevalent in COPD patients with associated MetS irrespective of gender, while chronic coronary syndrome, heart failure, and peripheral vascular disease reached the statistical limit only in the subgroup of male patients. Of the pulmonary comorbidities, OSA was predominantly present in patients with COPD and MetS (*p* < 0.001), whereas chronic oxygen therapy requirement was associated with a 3-fold higher percentage among male COPD patients without MetS (*p* = 0.033).

Table 3 compares anthropometric parameters, biological data, and functional respiratory parameters at baseline and at the 6-month follow-up. Abdominal circumference and FEV1 significantly improved in male patients with COPD and MetS (*p* < 0.005).

The number of patients with low-grade dyspnea (less than 2 on the mMRC scale) significantly increased at follow-up among male patients with and without MetS (*p* < 0.005).

**Figure 2:**
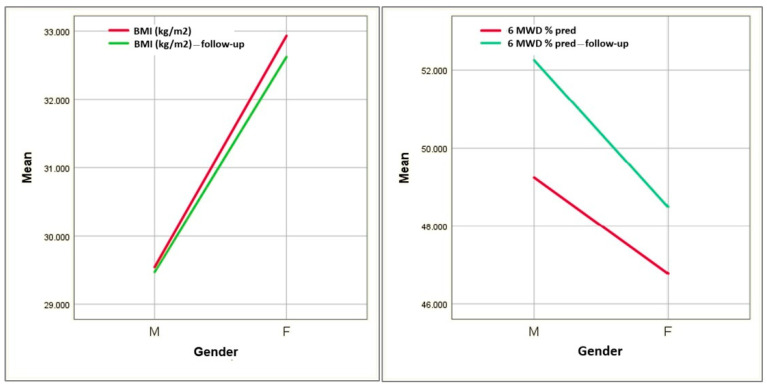
Evolution of BMI and distance at 6MWT depending on gender.

When analyzing our entire COPD group as a whole, we noted a significant improvement in 6MWT results. However, in the subgroup analysis, the change remained statistically significant only in males and especially in males without MetS (*p* < 0.005) (Figure 2).

Fasting glucose improved in our entire COPD group as a whole and remained statistically significant in male and female COPD patients with associated MetS. While HDL and triglycerides did not significantly vary at follow-up, LDL improved in males and females with COPD and remained statistically significant in female patients with COPD and associated MetS.

Average SBP values decreased in the COPD group as a whole, but especially in male patients with and without associated MetS. DBP values improved in our study group as a whole, as well as in patients without associated MetS, but not in males with COPD and MetS.

BMI was positively correlated with FEV1 and the FEV1/FVC ratio in all COPD patients and in the MetS subgroup. In the COPD group as a whole, the 6MWT results (m) were positively correlated with FEV1 and FVC. The correlation remained significant for FVC in COPD patients with and without MetS (Table 4).

Using multivariate statistical analysis (Table 5), we demonstrated that a one unit increase in BMI significantly augments TG values by 3.358 mg/dL and that each one unit increase in the number of pack-years leads to an increase in TG values by 0.791.

## 4. Discussion

COPD and MetS are conditions with an increasing incidence and an important impact on public health services. COPD patients with MetS have a lower quality of life, are at increased risk of exacerbation, and require more financial resources [59,60]. Numerous studies [60,61,62,63,64] have shown that subjects with COPD and MetS are younger than those without MetS, but there are also studies that have shown the opposite [65,66,67]. In our study, those with concomitant COPD-MetS were younger, with a mean age of 66.22 ± 8.528 years vs 68.64 ± 10.665 years.

Smoking is a risk factor for both COPD and the development of cardiovascular comorbidities such as stroke and coronary artery disease [68]. Furthermore, it is known that smoking can induce insulin resistance, and its association with systemic inflammation can lead to metabolic syndrome [69,70]. In our study, subjects with MetS have a lower pack-year smoking history, most of them being former smokers or even non-smokers. In this case, the presence of airflow obstruction could be secondary to passive smoking or exposure to respiratory toxins.

Similar to the results of Bermudez et al. [67], the presence of MetS was not correlated with the severity of airway obstruction in our study group. A meta-analysis of 22 studies and a total of 21,150 subjects with COPD highlighted the impact of obesity on lung function and cited an increased risk of mortality among subjects with low BMI compared with overweight or obese subjects [71]. A study in Copenhagen showed an inversely proportional relationship between the risk of death in COPD and BMI, with the risk of mortality increasing with decreasing BMI (RR 2.14 95% CI (1.18–3.89)) [72].

Complementary to BMI, abdominal circumference is a more accurate indicator used in many studies to indicate fat distribution [73]. Visceral fat, localized in the abdomen, increases the risk of developing a number of conditions with serious consequences for the patient’s health [74]. The accumulation of visceral fat is not only a risk factor for cardiovascular and metabolic diseases but can also increase the risk of sleep apnea, liver steatosis, Alzheimer’s disease, and even certain types of cancer [75]. Our study shows statistically significant results of abdominal circumference (*p* < 0.001), leading to an increased risk of pulmonary and systemic damage. More prospective studies [73,76,77] analyzed the relationship between BMI, abdominal circumference, and risk of lung damage. The results showed an increased risk in the presence of increased abdominal circumference. Furthermore, BMI affects lung function depending on the presence of increased abdominal circumference [78]. Cruthirds et al. [39] analyzed the link between increased abdominal girth and mood disorders in subjects with COPD. The results of their study showed that depression and anxiety can influence BMI and have an increased prevalence in subjects with larger abdominal circumference. Thus, increased abdominal girth could be a risk factor in the development of cognitive dysfunction and disorders such as depression and anxiety [39,79].

Concerning the GOLD stage, our results are similar to those in the literature, where most subjects with MetS present GOLD stage II. This is of major importance for the prognosis of the disease as it may indicate the impact of lifestyle, where subjects with COPD and MetS have an increased cardiovascular risk. It is also necessary to monitor patients in the long term, as there is a risk of early mortality through the development of cardiovascular complications.

The average BMI in patients with MetS was 32.57 ± 6.635 kg/m^2^ compared with those without MetS, whose average was 26.68 ± 5.488 kg/m^2^ (*p* < 0.001). Previous studies [80] have shown the link between increased BMI and the risk of developing MetS. In terms of long-term outcomes, it is known that exercise contributes to weight loss and decreases cardiovascular risk, but several studies [38,81,82,83,84,85] have shown that a low BMI in patients with COPD increases the risk of mortality. This prognosis is explained by the fact that BMI does not differentiate between muscle mass and fat mass, and in GOLD stage IV patients, a loss of muscle mass (sarcopenia) occurs [86,87,88]. On the other hand, subjects with obesity show a decrease in lung volume but have better lung function compared with those with low BMI GOLD stage IV COPD, where there is a high degree of hyperinflation [89]. The present study showed positive correlations in subjects with COPD and MetS between BMI and FEV1 (r = 0.389, *p* = 0.001) and the FEV1/FVC ratio (r = 0.508, *p* < 0.001), respectively. This may be consistent with the obesity paradox and a better prognosis in the early stages of the disease.

Of the metabolic syndrome criteria, abdominal circumference, glucose level, and lipid-lowering treatment showed statistically significant results. Some studies [90,91,92] mention that subjects with abdominal obesity and good muscle function show better results in the 6-min walk test compared with those with low muscle mass. In our study, subjects with MetS and COPD performed a lower perimeter walk compared with those without MetS and a lower distance at the 6-month assessment. Statistically significant correlations were observed in subjects without MetS between 6MWD (%) and FEV1 (r = 0.447, *p* = 0.022) and FVC (r = 0.416, *p* = 0.034) in subjects without MetS [93]. The 6-minute walk test is a simple test that can assess the risk of functional impairment. A cross-sectional study by Liwsrisakun et al. showed an increased risk of functional impairment in subjects with COPD who performed a perimeter walk of less than 300 m [90].

Although the link between impaired lung function and cardiovascular events and type 2 diabetes mellitus has been recognized, the association between impaired lung function and metabolic syndrome has not been comprehensively assessed in the United States (U.S.) population. A previous study aimed to explore the association between impaired lung function and metabolic syndrome in a nationally representative sample of men and women. The cross-sectional population-based study included 8602 participants aged 20–65 years in the Third National Health and Nutrition Examination Survey (NHANES III) [94]. The authors examined the relationship between the different features of metabolic syndrome and lung function, including forced vital capacity (FVC) and forced expiratory volume in 1 s (FEV1). After adjusting for potential confounders such as age, body mass index, inflammatory factors, medical condition, and smoking status, participants with more components of metabolic syndrome had lower predicted values of FVC and FEV1 (*p* for trend < 0.001 for both). Impaired pulmonary function was also associated with individual components of metabolic syndrome, such as abdominal obesity, high blood pressure, high triglycerides, and low high-density lipoprotein (HDL) cholesterol (*p* < 0.05 for all parameters). These results from the nationally representative sample of U.S. adults suggested that a greater number of features of metabolic syndrome is strongly associated with poorer FVC and FEV1. In clinical practice, more comprehensive management strategies to address subjects with metabolic syndrome and impaired lung function need to be developed and investigated.

The results of our study show elevated triglyceride values in women. Furthermore, a statistically significant correlation was observed between increased BMI and increased triglycerides. The presence of MetS causes increased triglycerides and higher values in smokers vs. non-smokers [20,93]. Numerous studies [95,96,97] mention decreased HDLc in subjects with COPD and MetS, but in our study, we observed optimal HDLc values. In addition, at the 6-month re-evaluation, patients showed higher HDLc values and decreased triglyceride levels. This shows the importance of self-management and strict adherence to medication. A study by Chen et al. reported a directly proportional relationship between the number of metabolic syndrome components and lung function. The authors found correlations between decreased FEV1, FCV, and decreased HDLc and increased TG and blood pressure in both sexes. The protective role of HDLc against atherosclerotic processes is well known, and unlike the other components of MetS, HDLc value correlates strongly with decreased lung function, especially in women [94].

The presence of high blood pressure, low HDLc, low HDLc, and high TG, together with systemic inflammation, are risk factors for low bone density and, over time, for the development of osteoporosis [98,99]. Of the 32 women included in our study, 25 had MetS. Symptoms of MetS in women over 50 years of age are more difficult to manage, and it is necessary to assess menopausal status since in pre- and postmenopausal women symptoms may be more pronounced. Furthermore, for good disease control, early diagnosis of COPD and screening for conditions such as depression and/or anxiety is necessary [100,101].

Some studies [102,103] mention that systemic inflammation contributes to increased triglyceride levels, and others have shown that increased IL-6 leads to decreased HDL-c. Dogra et al. mentioned the role of IL-6 in screening MetS in COPD subjects [104]. In our study, inflammatory markers were assayed, among which IL-6 was chosen, and higher values were observed in the MetS group. Moreover, in our study, subjects with concomitant COPD-MetS showed a higher degree of inflammation, with increased IL-6, IL-1β, and TNF-alpha values compared with those without MetS. Fabbri et al. proposed the investigation of pulmonary and systemic inflammation, with the addition of the concept of chronic systemic inflammatory syndrome in the diagnosis of COPD subjects [105], which may influence the development of comorbidities. Proinflammatory cytokines such as IL-1β or TNF-alpha are involved in the initiation and maintenance of systemic inflammation by increasing the production of adipokines that influence lipid and carbohydrate metabolism. The increased production of adipokines such as leptin or adiponectin leads to metabolic syndrome, insulin resistance, and the development of non-alcoholic fatty liver disease [28,106,107,108].

Diabetes mellitus, dyslipidemia, hypertension, ischemic heart disease, and congestive heart disease are frequent comorbidities, especially in male patients with concomitant COPD and MetS, contributing to increased morbi-mortality [16]. In our study, only male patients with concomitant COPD and MetS had a significantly higher prevalence of cardiovascular comorbidities. Overall, 85.1% of subjects with COPD and MetS in our study were on antihypertensive treatment. These results show an increased prevalence of hypertension compared with other studies. The prevalence of AH in the study by Lam et al. was 56.7% [109], and in the study by John Kennedy et al., it was 77.2% [97].

Our study was conducted during the SARS-CoV-2 pandemic, which led to the need to develop the concept of self-management. Thus, subjects were encouraged to learn about their disease, and, given that they were hospitalized in a respiratory rehabilitation ward, they were trained to perform respiratory rehabilitation techniques. The program was individualized, started in the hospital, and continued at home. In 2003, Bourbeau et al. [110] mentioned the importance of self-management in the management of the disease through adherence to pharmacological and non-pharmacological treatment, with the adoption of healthy eating behavior. Emotional support is also of major importance, with encouragement for patients to include activities that increase quality of life by reducing symptoms, increasing well-being, and belonging to a support group (family, friends) [110]. The definition of self-management is presented in a 2016 paper by Effing et al. [111], which mentioned the importance of the patient’s knowledge of the disease and self-management by increasing motivation, physical exercise, stress management, meditation, and also the knowledge of sleep hygiene for good quality sleep. Some studies have shown the benefit of self-management in subjects with COPD, a decrease in the number of exacerbations and emergency department presentations, and lower medical costs [34,112,113,114].

The limitations of our study were the small number of subjects and the single-center, observational design of this study, which did not allow for the investigation of cause–effect relationships.

## 5. Conclusions

MetS is a common comorbidity in patients with COPD and is associated with higher BMI, fasting glucose, and triglycerides but not with higher IL-1β, IL-6, IL-8, or TNF-alpha values. In COPD patients, triglyceride levels increase with BMI and the number of pack-years. A mixed pulmonary–cardiovascular rehabilitation intervention leads to a decrease in LDL in female COPD patients and to an improvement in abdominal circumference, FEV1, dyspnea, and blood pressure values in male patients with COPD; fasting glucose improved in all male and female COPD patients with associated MetS.

## Figures and Tables

**Table 1:** Comparative analyses of major characteristics between groups.

Parameters	Total (Mean ± SD)	COPD with MetS (n = 67) n (%) or Mean ± SD	COPD Without MetS (n = 33) n (%) or Mean ±SD	*p*-Value
Age, years	67.02 ± 9.303	66.22 ± 8.528	68.64 ± 10.665	0.155
Gender				0.105
Male	68 (68.0)	42 (62.7)	26 (78.8)	
Female	32 (32.0)	25 (37.3)	7 (21.2)	
BMI, kg/m^2^	30.63 ± 6.842	32.57 ± 6.635	26.68 ± 5.488	<0.001
BMI ≥ 25 kg/m^2^	79 (79.0)	62 (92.5)	17 (51.5)	<0.001
Abdominal circumference, cm	107.16 ± 14.673	110.93 ± 13.120	99.52 ± 14.871	<0.001
Smoking status				0.412
Never smoker	26 (26.0)	20 (29.9)	6 (18.2)	
Current smoker	25 (25.0)	15 (22.4)	10 (30.3)	
Former smoker	49 (49.0)	32 (47.8)	17 (51.5)	
Smoking history, pack-years	22.64 ± 20.244	21.28 ± 21.471	25.39 ± 17.477	0.196
GOLD classification				<0.001
1 (mild)	1 (1.0)	1 (1.5)	-	
2 (moderate)	54 (54.0)	35 (52.2)	19 (57.6)	
3 (severe)	33 (33.0)	29 (43.3)	4 (12.1)	
4 (very severe)	12 (12.0)	2 (3.0)	10 (30.3)	
FEV1, % (mean ± SD)	54.65 ± 15.459	56.69 ± 12.218	50.50 ± 20.121	0.411
FVC, % (mean ± SD)	67.77 ± 13.792	70.07 ± 10.966	63.11 ± 17.521	0.119
FEV1/FVC ratio (mean ± SD)	60.25 ± 10.048	61.18 ± 8.457	58.35 ± 12.621	0.681
IL-8, pg/mL	148.02 ± 143.419	138.84 ± 130.043	168.21 ± 170.406	0.589
TNF-alpha, pg/mL	16.84 ± 2.084	16.95 ± 2.479	16.614 ± 0.627	0.689
IL-6, pg/mL	4.28 ± 53.928	6.82 ± 62.685	1.33 ± 26.153	0.897
IL-1β, pg/mL	20.59 ± 88.310	30.79 ± 103.981	1.85 ± 24.089	0.060
Fasting glucose, mg/dL	111.40 ± 27.611	115.35 ± 28.653	103.39 ± 23.802	0.004
TG, mg/dL	132.12 ± 62.746	144.47 ± 66.898	107.04 ± 44.519	0.003
HDL cholesterol, mg/dL	57.80 ± 15.101	57.03 ± 15.416	59.37 ± 14.543	0.468
LDL cholesterol, mg/dL	119.35 ± 35.030	122.39 ± 35.853	113.16 ± 32.959	0.217
SpO2, %	84.367 ± 6.6543	84.105 ± 6.7374	85.100 ± 6.5285	0.478
Short-acting BD	92 (92.0)	63 (94.0)	29 (87.9)	0.434
Long-acting BD	95 (95.0)	63 (94.0)	32 (97.0)	1.000
Inhaled CS	48 (48.0)	34 (50.7)	14 (42.4)	0.433
Statins	57 (57.0)	44 (65.7)	13 (39.4)	0.013
Other hypolipidemic drugs	23 (23.0)	22 (32.8)	1 (3.0)	0.001
Oral hypoglycemic drugs	43 (43.0)	36 (53.7)	7 (21.2)	0.002
Insulin	7 (7.0)	6 (9.0)	1 (3.0)	0.420
Blood pressure lowering drugs	81 (81.0)	57 (85.1)	24 (72.7)	0.139
Beta-blockers	47 (47.0)	34 (50.7)	13 (39.4)	0.285
Psychiatric drugs	25 (25.0)	18 (26.9)	7 (21.2)	0.539

Variables described as mean ± standard deviation (SD) or as frequency (%). Abbreviations: COPD, chronic obstructive pulmonary disease; MetS, metabolic syndrome; BMI, body mass index; FEV1, forced expiratory volume in the first second; FVC, forced vital capacity, IL-8, interleukin-8; TNF-alpha, tumor necrosis factor-alpha; IL-6, interleukin-6; IL-1β, interleukin-1 beta; TG, triglyceride; HDL-C, high-density lipoprotein cholesterol; LDL-c, low-density lipoprotein cholesterol; SpO2 min, minimum peripheral oxygen saturation; Global Initiative for Chronic Obstructive Lung Disease (2024); BD, bronchodilator; CSs, corticosteroids.

**Table 2:** Comorbidities—gender differences in the two groups.

	Total n = 100	Men			Women		
		COPD with MetS (n = 42)	COPD Without MetS (n = 26)	*p*	COPD with MetS (n = 25)	COPD Without MetS (n = 7)	*p*
Diabetes, n (%)	49 (49.0)	27 (64.3)	6 (23.1)	0.001	15 (60.0)	1 (14.3)	0.033
Dyslipidemia, n (%)	69 (69.0)	33 (78.6)	9 (34.6)	<0.001	24 (96.0)	3 (42.9)	<0.001
Hypertension, n (%)	85 (85.0)	40 (95.2)	16 (61.5)	<0.001	23 (92.0)	6 (85.7)	0.536
Atrial fibrillation, n (%)	12 (12.0)	4 (9.5)	2 (7.7)	1.000	3 (12.0)	3 (42.9)	0.101
Chronic coronary syndrome, n (%)	64 (64.0)	31 (73.8)	11 (42.3)	0.012	18 (72.0)	4 (57.1)	0.454
Heart failure, n (%)	57 (57.0)	30 (71.4)	11 (42.3)	0.023	12 (48.0)	4 (57.1)	0.669
Peripheral vascular disease, n (%)	31 (31.0)	20 (47.6)	5 (19.2)	0.022	5 (20.0)	1 (14.3)	0.732
Sleep apnea, n (%)	49 (49.0)	27 (64.3)	5 (19.2)	<0.001	14 (56.0)	3 (42.9)	0.538
Depression, n (%)	50 (50.0)	22 (52.4)	11 (42.3)	0.462	13 (52.0)	4 (57.1)	0.810
Anxiety, n (%)	39 (39.0)	20 (47.6)	7 (26.9)	0.127	9 (36.0)	3 (42.9)	0.740
Pulmonary hypertension, n (%)	25 (25.0)	13 (31.0)	3 (11.5)	0.083	7 (28.0)	2 (28.6)	0.976
Oxygen users, n (%)	18 (18.0)	5 (11.9)	9 (34.6)	0.033	4 (16.0)	-	0.258

Data are presented as frequency (%). Abbreviations: COPD, chronic obstructive pulmonary disease; MetS, metabolic syndrome.

**Table 3:** Differences between anthropometric, biological, and functional respiratory parameters at the 6-month assessment compared to the initial evaluation.

	Total (n = 100)			COPD with MetS (n = 67)			COPD Without MetS (n = 33)		
	Baseline	Follow-Up	*p*	Baseline	Follow-Up	*p*	Baseline	Follow-Up	*p*
BMI, kg/m^2^ (mean ± SD)									
Total	30.63 ± 6.842	30.48 ± 6.703	0.469	32.57 ± 6.635	32.24 ± 6.514	0.254	26.68 ± 5.488	26.91 ± 5.642	0.602
Men	29.54 ± 5.851	29.47 ± 5.805	0.691	31.65 ± 5.476	31.35 ± 5.523	0.372	26.15 ± 4.811	26.43 ± 4.975	0.492
Women	32.93 ± 8.214	32.62 ± 7.980	0.555	34.12 ± 8.113	31.35 ± 5.523	0.523	28.68 ± 7.635	28.66 ± 7.879	1.000
Weight, kg (mean ± SD)									
Total	85.69 ± 8.212	85.42 ± 18.164	0.562	90.21 ± 16.894	89.48 ± 16.911	0.231	76.52 ± 17.548	77.18 ± 18.067	0.328
Men	87.93 ± 18.073	87.59 ± 17.740	0.541	94.36 ± 15.389	93.26 ± 15.166	0.130	77.54 ± 17.473	78.42 ± 18.023	0.297
Women	80.94 ± 17.861	80.81 ± 18.471	0.887	83.24 ± 17.309	83.12 ± 18.065	0.915	72.71 ± 18.679	72.57 ± 18.867	0.846
Abdominal circumference, cm (mean ± SD)									
Total	107.16 ± 14.673	105.80 ± 14.649	0.039	110.93 ± 13.120	108.91 ± 13.588	0.010	99.52 ± 14.871	99.48 ± 14.892	0.980
Men	108.21 ± 14.377	106.49 ± 13.85	0.070	113.48 ± 11.458	110.57 ± 11.972	0.016	99.69 ± 14.718	99.88 ± 14.342	0.900
Women	104.94 ± 15.276	104.34 ± 16.356	0.189	106.64 ± 14.784	106.12 ± 15.81	0.312	98.86 ± 16.618	98.00 ± 17.954	0.418
FEV1, % (mean ± SD)									
Total	54.65 ± 15.459	56.07 ± 14.933	0.001	56.69 ± 12.218	58.54 ± 12.418	0.001	50.50 ± 20.121	51.04 ± 18.246	0.213
Men	51.29 ± 16.154	53.11 ± 15.659	0.001	54.24 ± 11.82	56.35 ± 12.151	0.004	46.52 ± 20.782	47.87 ± 19.203	0.060
Women	61.8 ± 11.029	62.35 ± 11.053	0.445	60.82 ± 11.983	62.22 ± 12.223	0.170	65.29 ± 5.992	62.83 ± 5.771	0.345
FVC, % (mean ± SD)									
Total	67.77 ± 13.792	68.51 ± 13.485	0.463	70.07 ± 10.966	70.69 ± 11.155	0.850	63.11 ± 17.521	64.11 ± 16.613	0.318
Men	64.77 ± 14.404	65.59 ± 13.964	0.699	67.87 ± 10.634	67.96 ± 10.61	0.492	59.76 ± 18.12	61.76 ± 17.698	0.196
Women	74.16 ± 9.856	74.73 ± 10.036	0.462	73.76 ± 10.719	75.26 ± 10.729	0.326	75.57 ± 6.268	72.81 ± 7.383	0.753
FEV1/FVC ratio (mean ± SD)									
Total	60.25 ± 10.048	61.58 ± 9.532	0.001	61.18 ± 8.457	62.41 ± 8.576	0.008	58.35 ± 12.621	59.88 ± 11.179	0.031
Men	58.33 ± 10.581	59.67 ± 9.927	0.010	59.64 ± 8.279	60.73 ± 8.525	0.062	56.22 ± 13.420	57.97 ± 11.832	0.062
Women	64.32 ± 7.436	65.63 ± 7.232	0.019	63.77 ± 8.275	65.24 ± 8.05	0.051	66.29 ± 2.446	67.00 ± 2.859	0.204
mMRC dyspnea scale, n (%)									
≤2									
Total	29 (29.0)	60 (60.0)	<0.001	20 (29.9)	42 (62.7)	<0.001	9 (27.3)	18 (54.5)	0.004
Men	16 (23.5)	40 (58.8)	<0.001	9 (21.4)	25 (59.5)	<0.001	7 (26.9)	15 (57.7)	0.008
Women	13 (40.6)	20 (62.5)	0.065	11 (44.0)	17 (68.0)	0.109	2 (28.6)	3 (42.9)	1.000
>2									
Total	71 (71.0)	40 (40.0)	<0.001	47 (70.1)	25 (37.3)	<0.001	24 (72.7)	15 (45.5)	<0.001
Men	52 (76.5)	28 (41.2)	<0.001	33 (78.6)	17 (40.5)	<0.001	19 (73.1)	11 (42.3)	<0.001
Women	19 (59.4)	12 (37.5)	0.081	14 (56.0)	8 (32.0)	.	5 (71.4)	4 (57.1)	0.042
CAT score, n (%)									
≤10									
Total	3 (3.0)	5 (5.0)	0.625	2 (3.0)	4 (6.0)	0.625	1 (3.0)	1 (3.0)	1.000
Men	1 (1.5)	1 (1.5)	1.000	-	-	-	1 (3.8)	1 (3.8)	1.000
Women	2 (6.3)	4 (12.5)	0.625	2 (8.0)	4 (16.0)	0.625	-	-	-
>10									
Total	97 (97.0)	95 (95.0)	0.772	65 (97.0)	63 (94.0)	0.982	32 (97.0)	32 (97.0)	0.158
Men	67 (98.5)	67 (98.5)	1.000	42 (100.0)	42 (100.0)	1.000	25 (96.2)	25 (96.2)	0.158
Women	30 (93.8)	28 (87.5)	0.601	23 (92.0)	21 (84.0)	0.189	7 (100.0)	7 (100.0)	1.000
6 MWD, m (mean ± SD)									
Total	220.76 ± 167.231	262.01 ± 122.633	0.003	215.50 ± 163.246	251.54 ± 118.605	0.049	231.43 ± 177.147	282.96 ± 130.146	0.016
Men	230.41 ± 173.533	270.34 ± 130.765	0.007	224.19 ± 166.834	258.53 ± 123.463	0.068	240.47 ± 186.782	288.33 ± 142.368	0.039
Women	200.24 ± 153.583	244.36 ± 103.553	0.203	200.91 ± 159.319	240.35 ± 112.583	0.365	197.86 ± 142.591	260.40 ± 61.080	0.345
6 MWD, % pred (mean ± SD)									
Total	40.53 ± 32.418	48.45 ± 26.365	0.005	39.09 ± 31.517	45.94 ± 25.533	0.053	43.47 ± 34.486	53.48 ± 27.781	0.019
Men	41.78 ± 32.95	49.24 ± 26.746	0.017	40.84 ± 31.811	47.43 ± 24.996	0.137	43.29 ± 35.301	52.01 ± 29.634	0.050
Women	37.89 ± 31.608	46.79 ± 26.001	0.149	36.15 ± 31.441	43.58 ± 26.850	0.268	44.11 ± 33.911	59.64 ± 19.335	0.225
Glucose, mg/dL (mean ± SD)									
Total	111.40 ± 27.611	103.9 ± 23.973	0.001	115.34 ± 28.653	105.49 ± 22.09	0.001	103.39 ± 23.802	100.66 ± 27.484	0.654
Men	111.53 ± 27.999	104.68 ± 24.05	0.040	117.39 ± 33.256	108.90 ± 26.737	0.048	102.07 ± 11.680	97.85 ± 17.301	0.420
Women	111.13 ± 27.205	102.24 ± 24.105	0.008	111.91 ± 18.678	99.77 ± 8.139	0.001	108.33 ± 49.162	111.09 ± 51.190	0.499
LDL-C, mg/dL, (mean ± SD)									
Total	119.35 ± 35.030	110.12 ± 31.172	0.002	122.39 ± 35.853	113.31 ± 34.187	0.013	113.16 ± 32.959	103.63 ± 23.040	0.059
Men	115.99 ± 34.847	108.43 ± 32.171	0.026	118.61 ± 38.460	111.80 ± 36.573	0.163	111.74 ± 28.252	102.98 ± 23.023	0.065
Women	126.49 ± 34.884	113.70 ± 29.098	0.030	128.74 ± 30.675	115.85 ± 30.303	0.040	118.44 ± 49.262	106.04 ± 24.778	0.398
HDL-C, mg/dL (mean ± SD)									
Total	57.80 ± 15.101	59.43 ± 12.363	0.204	57.03 ± 15.416	59.06 ± 12.031	0.238	59.37 ± 14.543	60.18 ± 13.170	0.645
Men	56.83 ± 14.700	57.82 ± 12.138	0.504	55.85 ± 14.096	57.41 ± 10.824	0.436	58.42 ± 15.780	58.47 ± 14.212	0.983
Women	59.86 ± 15.961	62.86 ± 12.322	0.240	59.01 ± 17.538	61.83 ± 13.608	0.382	62.89 ± 8.498	66.54 ± 4.855	0.163
TG, mg/dL (mean ± SD)									
Total	132.12 ± 62.746	127.77 ± 54.703	0.732	144.47 ± 66.898	139.63 ± 58.267	0.987	107.04 ± 44.519	103.69 ± 36.916	0.525
Men	130.86 ± 68.359	125.49 ± 57.576	0.973	146.02 ± 77.456	139.25 ± 63.487	0.827	106.37 ± 40.918	103.25 ± 37.97	0.737
Women	134.80 ± 49.626	132.64 ± 48.537	0.570	141.87 ± 45.243	140.28 ± 49.500	0.753	109.56 ± 59.873	105.33 ± 35.455	0.398
SBP, mmHg (mean ± SD)									
Total	136.18 ± 17.998	131.18 ± 13.037	0.001	137.01 ± 19.072	132.48 ± 13.219	0.016	134.48 ± 15.732	128.55 ± 12.44	0.014
Men	138.90 ± 19.002	131.07 ± 13.769	0.000	140.74 ± 20.187	131.86 ± 14.598	0.001	135.92 ± 16.866	129.81 ± 12.487	0.022
Women	130.41 ± 14.264	131.41 ± 11.531	0.906	130.76 ± 15.463	133.52 ± 10.709	0.699	129.14 ± 9.616	123.86 ± 11.964	0.271
DBP, mmHg (mean ± SD)									
Total	81.45 ± 9.950	78.70 ± 8.796	0.001	81.82 ± 10.521	79.54 ± 9.29	0.033	80.70 ± 8.780	77.00 ± 7.542	0.009
Men	82.69 ± 10.076	79.40 ± 7.81	0.004	83.24 ± 11.078	80.36 ± 8.479	0.080	81.81 ± 8.338	77.85 ± 6.442	0.011
Women	78.81 ± 9.282	77.22 ± 10.579	0.158	79.44 ± 9.238	78.16 ± 10.554	0.220	76.57 ± 9.813	73.86 ± 10.761	0.500

Data are presented as mean ± standard deviation (SD) or as frequency (%). Abbreviations: COPD, chronic obstructive pulmonary disease; MetS, metabolic syndrome; BMI, body mass index; FEV1, forced expiratory volume in the first second; FVC, forced vital capacity, mMRC dyspnea scale, Medical Research Council scale; CAT score, COPD Assessment Test score; 6 MWD, 6-min walk test; LDL-c, low-density lipoprotein cholesterol; HDL-C, high-density lipoprotein cholesterol; TG, triglyceride; SBP, systolic blood pressure; DBP, diastolic blood pressure; Global Initiative for Chronic Obstructive Lung Disease (2024).

**Table 4:** Correlations between spirometry results and BMI, and between spirometry and 6MWT results.

	Total				COPD with MetS				COPD Without MetS			
Spirometry Parameters			95% Confidence Interval				95% Confidence Interval				95% Confidence Interval	
	r	*p*	Lower Limit	Upper Limit	r	*p*	Lower Limit	Upper Limit	r	*p*	Lower Limit	Upper Limit
BMI												
Baseline												
FEV1 (%)	0.342	<0.001	0.157	0.505	0.389	0.001	0.164	0.575	0.198	0.270	−0.156	0.507
FVC %	0.171	0.089	−0.026	0.355	0.071	0.570	−0.173	0.306	0.114	0.526	−0.238	0.440
FEV1/FVC	0.379	<0.001	0.198	0.536	0.508	<0.001	0.305	0.667	0.149	0.408	−0.205	0.468
Follow-up												
FEV1 (%)	0.333	<0.001	0.146	0.497	0.361	0.003	0.132	0.553	0.149	0.409	−0.205	0.468
FVC %	0.223	0.026	0.028	0.402	0.127	0.304	−0.116	0.357	0.202	0.259	−0.151	0.510
FEV1/FVC	0.349	<0.001	0.164	0.510	0.484	<0.001	0.276	0.649	0.062	0.734	−0.288	0.396
Distance at 6MWT (m)												
Baseline												
FEV1 (%)	0.289	0.010	0.071	0.481	0.227	0.106	−0.049	0.470	0.447	0.022	0.072	0.711
FVC %	0.343	0.002	0.131	0.526	0.370	0.007	0.108	0.584	0.416	0.034	0.034	0.692
FEV1/FVC	0.144	0.209	−0.081	0.355	0.153	0.279	−0.125	0.409	0.171	0.405	−0.232	0.523
Follow-up												
FEV1 (%)	0.185	0.065	−0.012	0.368	0.104	0.403	−0.140	0.336	0.344	0.050	0.001	0.615
FVC %	0.206	0.040	0.010	0.387	0.184	0.137	−0.059	0.406	0.289	0.103	−0.060	0.575
FEV1/FVC	0.132	0.190	−0.066	0.320	0.079	0.525	−0.164	0.313	0.234	0.191	−0.119	0.534

Abbreviations: r Pearson correlation coefficient; COPD, chronic obstructive pulmonary disease; MetS, metabolic syndrome; BMI, body mass index; FEV1, forced expiratory volume in the first second; FVC, forced vital capacity, 6MWT 6 minute walk test.

**Table 5:** Multivariate statistical analysis.

Triglycerides		Unstandardized Coefficient		*p*	95% Confidence Interval (B)
		B (Effect)	Std. Error		
	(Constant)	28.013	54.651	0.609	−80.513 ÷ 136.538
	Age, years	−0.710	0.649	0.277	−2.000 ÷ 0.579
	Gender	12.817	17.735	0.472	−22.401 ÷ 48.035
	BMI (kg/m^2^)	3.358	0.963	<0.001	1.447 ÷ 5.270
	MetS	17.433	13.332	0.194	−9.042 ÷ 43.908
	Smoking	1.889	9.509	0.843	−16.995 ÷ 20.773
	Smoking history, pack-years	0.792	0.373	0.036	0.053 ÷ 1.532

Data were calculated using multiple linear regression. Abbreviations: Std. error, standard error; BMI, body mass index; MetS, metabolic syndrome.

## Data Availability

Data is available from the corresponding author upon request.
